# The Intimate Relationship among EMT, MET and TME: A T(ransdifferentiation) E(nhancing) M(ix) to Be Exploited for Therapeutic Purposes

**DOI:** 10.3390/cancers12123674

**Published:** 2020-12-07

**Authors:** Ralf Hass, Juliane von der Ohe, Hendrik Ungefroren

**Affiliations:** 1 Biochemistry and Tumor Biology Lab, Department of Obstetrics and Gynecology, Hannover Medical School, 30625 Hannover, Germany; ohe.juliane.von.der@mh-hannover.de; 2 First Department of Medicine, UKSH, Campus Lübeck, 23538 Lübeck, Germany; hendrik.ungefroren@uksh.de; 3 Department of General Surgery, Visceral, Thoracic, Transplantation and Pediatric Surgery, UKSH, Campus Kiel, 24105 Kiel, Germany

**Keywords:** tumor heterogeneity, plasticity, epithelial-mesenchymal transition, retrodifferentiation, cancer stem cells, transdifferentiation, cancer cell fusion, post-hybrid selection process, mesenchymal stroma/stem-like cells, tumor therapy

## Abstract

**Simple Summary:**

Intratumoral heterogeneity is considered the major cause of drug resistance and hence treatment failure in cancer patients. Tumor cells are known for their phenotypic plasticity that is the ability of a cell to reprogram and change its identity to eventually adopt multiple phenotypes. Tumor cell plasticity involves the reactivation of developmental programs, the acquisition of cancer stem cell properties and an enhanced potential for retro- or transdifferentiation. A well-known transdifferentiation mechanism is the process of epithelial-mesenchymal transition (EMT). Current evidence suggests a complex interplay between EMT, genetic and epigenetic alterations, and various signals from the tumor microenvironment (TME) in shaping a tumor cell’s plasticity. The vulnerabilities exposed by cancer cells when residing in a plastic or stem-like state have the potential to be exploited therapeutically, i.e., by converting highly metastatic cells into less aggressive or even harmless postmitotic ones.

**Abstract:**

Intratumoral heterogeneity is considered the major cause of drug unresponsiveness in cancer and accumulating evidence implicates non-mutational resistance mechanisms rather than genetic mutations in its development. These non-mutational processes are largely driven by phenotypic plasticity, which is defined as the ability of a cell to reprogram and change its identity (phenotype switching). Tumor cell plasticity is characterized by the reactivation of developmental programs that are closely correlated with the acquisition of cancer stem cell properties and an enhanced potential for retrodifferentiation or transdifferentiation. A well-studied mechanism of phenotypic plasticity is the epithelial-mesenchymal transition (EMT). Current evidence suggests a complex interplay between EMT, genetic and epigenetic alterations, and clues from the tumor microenvironment in cell reprogramming. A deeper understanding of the connections between stem cell, epithelial–mesenchymal, and tumor-associated reprogramming events is crucial to develop novel therapies that mitigate cell plasticity and minimize the evolution of tumor heterogeneity, and hence drug resistance. Alternatively, vulnerabilities exposed by tumor cells when residing in a plastic or stem-like state may be exploited therapeutically, i.e., by converting them into less aggressive or even postmitotic cells. Tumor cell plasticity thus presents a new paradigm for understanding a cancer’s resistance to therapy and deciphering its underlying mechanisms.

## 1. Introduction

In modern cancer medicine, the development of therapeutic resistance is the cause of treatment failure and disease recurrence and thus a major challenge for the clinical management of cancer. The underlying mechanism considered responsible for different response rates to drug treatment is inter- and intratumoral heterogeneity. Intertumor heterogeneity designates the variability across different tumors of different patients, while intratumor heterogeneity results from variability within an individual tumor. Cancer cells evolve and constitute heterogeneous populations that fluctuate in space and time and are subject to selection pressures generating intratumor heterogeneity. Tumors arising from the same type of cell in different patients may share some common features but are never identical. Intertumor heterogeneity sometimes also refers to clusters of malignant cells within the same patient such as primary tumor, lymph node metastases, distant metastases, and circulating tumor cells (CTCs) [1].

In many cancer types, intertumoral heterogeneity manifests besides classical histomorphological traits, epithelial versus mesenchymal differentiation, in distinct molecular signatures. Cancers that appear morphologically similar often have dramatically different clinical features, respond variably to therapy and have a range of clinical outcomes. Mesenchymal subtypes are often associated with drug resistance and a worse prognosis. A prime example is represented by pancreatic ductal adenocarcinoma (PDAC). Disease stratification efforts of PDAC have revealed the existence of several distinct subtypes with unique molecular signatures and therapeutic vulnerabilities. Recent data have shown that certain subtypes of PDAC are completely resistant to standard chemotherapies. Hence, defining subtype-specific features will permit patient stratification and precise molecularly-based therapeutic interventions [2]. The ability to predict optimal therapeutic strategies in advance of treatment improves overall patient outcomes, minimizing treatment-related morbidity and cost. In the meanwhile, this has become routine for many cancer types, but not for PDAC. However, accumulating molecular data are defining subgroups in pancreatic cancer with distinct biology and potential subtype-specific therapeutic vulnerabilities [3].

Intratumor heterogeneity refers to the presence of different cell subpopulations within a given tumor sample. Tumors are composed of a mosaic of cell subpopulations, which are often referred to as tumor clones. They may differ in cell morphology, the spectrum of genetic and epigenetic alterations, metabolism, proliferative capacity, and metastatic potential. Indeed, intratumor heterogeneity exists at several levels, including (i) genetic and epigenetic variability among different cells in the same tumor, (ii) extrinsic cell-cell and cell-matrix interactions and iii) the availability of nutrients, oxygen, and growth factors within the tumor microenvironment (TME), eventually leading to phenotype diversification, a phenomenon termed “positional heterogeneity”. Finally, tumors are constantly changing under various selective pressures, i.e., medications, nutrition, and hormonal status, as well as the cancer drugs themselves, all of which can influence the evolution of a tumor (temporal heterogeneity) [4].

To explain tumor heterogeneity, two models have been postulated, the cancer stem cell model and the clonal evolution model [4]. The first one proposes that a small subpopulation of cells with self-renewal potential drive tumor progression and their differentiation may generate the variability observed within a tumor. These cells also account for tumor relapse/recurrence, the repopulation of a tumor following treatment. The second model proposes that premalignant or malignant cells accumulate genetic and epigenetic changes over time driven essentially by their inherent genomic instability. These changes eventually confer selective advantages on the cell that are subsequently selected in a Darwinian-like evolutionary process. The cells continue to accumulate (epi)genetic changes, thereby driving the diversification of the tumor and leading to the phenotypes observed in an advanced cancer such as hyper-proliferation, invasion/metastasis, apoptosis/drug resistance, and immune evasion. The genotypic and phenotypic variability of tumors can have important consequences for diagnosis, prognosis, and treatment. However, proper assessment of the heterogeneity present within a given tumor sample is difficult for the following reasons. Analysis of a single biopsy specimen may not be an accurate representation of the entire tumor of a patient as it is unlikely to capture the full spectrum of distinct clones present within this tumor. Thus, heterogeneity can prevent reliable prognosis due to biopsy sampling bias. As a consequence, therapy design based on a single biopsy site may not result in response to all areas of the tumor and the cancer cells that escape treatment eventually cause tumor recurrence. In addition, a biopsy only reflects the actual stage of tumor development and there is no standard way to predict how a tumor will evolve in the future. Repeated biopsy sampling of the tumor at the time of disease progression or during the course of a particular therapy to follow-up genetic, epigenetic or metabolic alterations has only lately becoming routine practice for some but not all cancers.

Pre-existing or acquired therapeutic resistance refers to genotypic and/or phenotypic changes within the tumor prior to or during therapy that favor the natural selection of drug-tolerant clones. Subsets of cells that manage to survive conventional therapy drive the relapse of the tumor, which are often more resistant to treatment and hence more aggressive than their ancestors. A frequently observed feature of malignant carcinomas is the partial loss of epithelial traits and the gain of certain mesenchymal ones by a genetic program termed epithelial-to-mesenchymal transition (EMT). When cells undergo an EMT they can acquire several abilities, i.e., to disseminate, to adopt stem-like or tumor-initiating capacity, to colonize distant sites in the body, and to become drug or therapy resistant. Recent work has revealed a great variability of the EMT program with complete and incomplete forms (see Section 3) the specific forming of which depends on tissue context and stage of tumor progression [5]. Numerous methods have been developed to detect tumor heterogeneity at the genetic, epigenetic, and phenotypic level [6]. These include multi-regional sampling (single biopsies or fine needle aspirates), cytological assays, immunohistochemistry, in situ hybridization, and omic-based technologies, i.e., whole genome and next-generation sequencing as well as single cell sequencing. Liquid biopsies based on cell-free circulating tumor DNA carrying tumor-specific genetic or epigenetic alterations, and CTCs provide a new approach to circumvent the challenges of spatial heterogeneity [6]. A novel method, termed MAPit-patch, uses multiplex amplification of targeted sequences from genomic DNA followed by next-generation bisulfite sequencing. It allows for a highly scalable and simultaneous mapping of chromatin accessibility and DNA methylation on single molecules [7]. The advent of deep sequencing technology has revealed a greater complexity of distinct genotypes and phenotypes that drive the biological behavior of cancer.

## 2. Cell Plasticity as Basis of Intratumoral Heterogeneity and Drug Resistance

The phenomenon of cellular plasticity—the ability of cells to change their phenotype in a reversible fashion—is involved in post-injury tissue repair and regeneration, as well as in epithelial homeostasis [8,9,10]. Cellular plasticity also plays a crucial role in the initiation and progression of multiple pathologies, including cancer [8,10,11,12]. Cell plasticity can partially explain the intratumoral heterogeneity with cancer cells exhibiting variable degrees of phenotypic interconversion between drug-susceptible and drug-refractory states [13].

**Figure 1:**
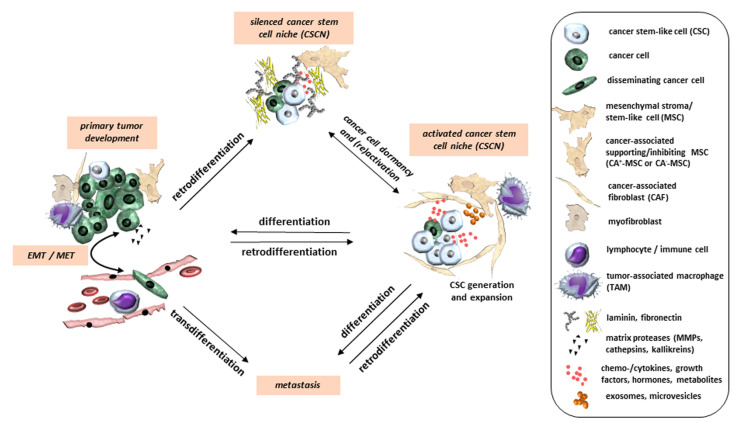
Different developmental states within a tumor entity determine the degree of tumor cell heterogeneity. Switches between these states can occur via different programs: (1) retrodifferentiation along a more immature or stem-like cancer cell phenotype; (2) transdifferentiation along a cancer cell phenotype with altered physiological and functional properties; (3) differentiation along a more maturated cancer cell phenotype with altered tumorigenicity and metastatic capacity (adapted from [45]).

Although an initial successful response to chemotherapy regimens, radiation treatments or targeted immunotherapies is often achieved, tumor recurrence is frequently observed by the development of new metastatic lesions. These residual cancer cells have apparently survived first-line therapies due to pre-existing or acquired resistance. The cause of this persisting minimal residual disease following cancer therapy may be mediated by various forms of phenotypic switching of drug-tolerant cancer cells. Moreover, these treatment-resistant cancer cells are regularly more aggressive than their therapy-tolerant counterparts, representing a major challenge for successful therapeutic interventions. Since the cellular differentiation state of the tumor often determines its response to therapy, it is not surprising that these tumors often present with phenotypes and molecular features of either stem cell, retrodifferentiated/dedifferentiated or transdifferentiated states, suggestive of cell plasticity [13,14,15,16]. Retrodifferentiation and dedifferentiation are often used interchangeably and describe expression patterns that were reversed from a differentiated phenotype to a precursor cell or stem cell level [17,18]. For instance, human neoplastic hepatocytes can retrodifferentiate to cancer stem-like cells (CSCs) [19]. Moreover, phorbol ester can induce in human acute promyelocytic leukemia (APL) cells an alternating differentiation and retrodifferentiation program [20]. Cells can also directly switch from one differentiated phenotype to another without passing through a pluripotent state, a process termed transdifferentiation [18,21] (Figure 1). For instance, based upon incomplete bile duct development or cell injury, hepatocytes can transdifferentiate into mature ductal biliary epithelial cells and form persistently functional bile ducts [22]. In case of de novo formation of the biliary system, previous work has demonstrated the involvement of transforming growth factor-β (TGF-β) in the transdifferentiation program of hepatocytes [23]. As for regeneration of normal tissues transdifferentiation can also play an important role in cancer cell plasticity and tumor development [24].

Other tumor-associated cellular events that contribute to increasing heterogeneity with clinical evidences include cell fusion [25]. Leucocyte-cancer cell fusion, particularly fusion of tumor-associated macrophages (TAMs) with cancer cells produces different hybrid cancer populations that express genetic and phenotypic characteristics of both parental cells [26,27,28,29]. Other cellular partners of cancer cell fusion are represented by mesenchymal stroma/stem-like cells (MSCs) and carcinoma-associated fibroblasts (CAFs) [30], eventually resulting in hybrid cancer populations with elevated [31,32] or reduced tumorigenicity [33,34]. In response to increased chromosomal instability in fused multinucleated giant cells a PHSP (post hybrid selection process) enables survival of a genetically stabilized phenotype [35]. Reprogramming during a PHSP increases tumor plasticity and can also contribute to the generation of CSCs (Figure 1). Hence, cancer cells may use various reprogramming capabilities to adjust to and sometimes escape from effects of therapies.

Induction of the EMT, a process that can increase stemness and tumor heterogeneity, likely contributes to resistance to chemotherapy, irradiation or immunotherapy. In general, resistance to therapy in solid tumors of breast, lung, pancreas and melanoma [36,37,38] is commonly associated with a mesenchymal state rather than an epithelial state [37,39,40,41].

The acquisition of therapy resistance has traditionally been considered to result from genetic mutations in the genome of cancer cells. However, accumulating evidence implicates a key role of non-mutation-based resistance mechanisms that result in tumor cell plasticity. These mechanisms render tumor cells refractory to the drug-targeted pathway, thereby facilitating tumor cell survival and growth. The differentiation state of a tumor is thus a key determinant of its therapeutic sensitivity [42]. Following retrodifferentiation, carcinoma cells acquire both of these critical malignant traits—metastasis and resistance—to a wide spectrum of chemotherapeutic drugs. Consistent with these findings in experimental models, high tumor grade, invasiveness, and survival of the cancer cells within the circulation correlate with poor response to chemotherapy [43,44].

Yet other cellular programs include a hybrid or partial (p), or complete (c) EMT with the generation of intermediate epithelilial/mesenchymal (E/M) cells and disseminated CTCs following trans-endothelial migration. Vice versa, these cell types can be reverted by a MET (mesenchymal-epithelial transition) after attachment to distal organs and tissues for initiation of metastatic outgrowth [46].

Radioresistance, chemoresistance, and the acquisition of a retrodifferentiated state are achieved by overexpression of certain transcription factors (TFs) associated with EMT or metaplasia and/or by the reactivation of stemness-related genes [47,48]. A retrodifferentiated state causes metabolic changes that impair pro-drug activation or drug uptake [48]. For instance, different breast cancer (BC) subtypes, which vary in tumor growth, drug sensitivity, and metastatic capacity can redevelop from retrodifferentiated breast CSCs. More specifically, the distinct cells of origin of BC subtypes are linked to specific genetic or epigenetic alterations following retro- or transdifferentiation which can mutually convert between basal and luminal cells. These events along intermediate mammary stem cell phenotypes suggest heterogeneous BC populations that are difficult to eliminate at the clinical level [49].

Individual cancer cells evolve with increasing genetic and phenotypic heterogeneity to a hierarchical organization whereby CSCs represent the top endowed with self-renewal capacity. The concept of tumor cell plasticity is also intimately connected to the reactivation of developmental programs that are closely correlated with EMT and transdifferentiation potential during drug exposure [50]. The impressive ability of tumor cells to switch their identities or phenotypes and stem cell state transitions may play a fundamental role in treatment escape.

## 3. EMT as a Transdifferentiation Process

**Figure 2:**
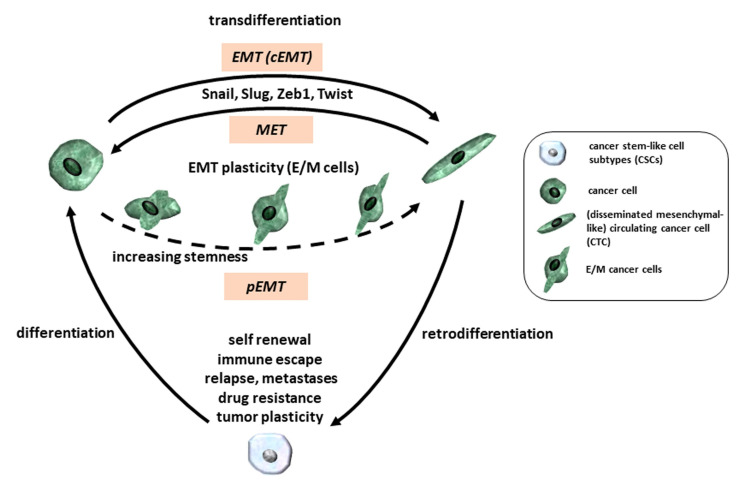
Various forms of EMT including complete EMT (cEMT) and partial EMT (pEMT) can be induced by different transcription factors including Snail, Slug, Zeb1, and Twist to switch epithelial and mesenchymal phenotypes in corresponding directions and to enable formation of multiple CSC subpopulations.

In addition to retrodifferentiation, transdifferentiation also contributes to tumor cell plasticity and thus tumor progression and metastasis (Figure 1). EMT is considered a transdifferentiation process that has been identified as a major contributing and well-studied mechanism to tumor cell phenotypic plasticity (Figure 2). The programs of EMT and MET, are involved in controlling a diverse array of physiologic processes like vertebrate embryonic development, wound healing, and tissue repair, but also various pathological events, such as fibrosis and tumor invasion and metastasis [51,52,53,54] in both, normal and neoplastic cells [40,55]. EMT is characterized by the loss of apico-basal polarity, rearrangements in the cytoskeleton and the acquisition of mesenchymal gene expression signatures [15]. It is generally governed by several EMT-associated TFs, such as Snail, Slug, zinc finger E-box-binding 1/2 (Zeb1/2) and Twist, microRNAs (miRs), or splicing factors in response to multiple signaling pathways, such as those of TGF-β, Wnt, Notch, and Ras-mitogen-activated protein kinase (Figure 2). Notably, Snail and Zeb act together with miR-34 and miR-200 in two double-negative feedback loops with the Snail/miR-34 regulatory loop preferentially participating in the initial phase of EMT in epithelial cells and the miR-200/Zeb loop controlling the transition to and maintenance of the mesenchymal state [56,57,58].

The EMT programs also promote CSC stemness in many epithelial tissues. Their activation is associated with the acquisition of stem-like characteristics such as enhanced colony formation in vitro and enhanced tumorigenesis in vivo [40] in different cancers [40,59,60,61]. EMT is thought to be regulated primarily at the transcriptional level through the activity of EMT-TFs. The majority of cancer cells does not undergo a cEMT, but rather adopt distinct features of mesenchymal cells, while maintaining some epithelial characteristics, resulting in intermediate cell states between the pure epithelial and the pure mesenchymal state. This phenomenon is referred to as pEMT (Figure 2). Programs driving EMT in physiological contexts, e.g., in a lineage-labeled mouse model of PDAC, in addition, revealed that carcinoma cells can lose their epithelial program through different mechanisms, which are associated with distinct modes of invasion and dissemination. The pEMT is dominated by protein internalization and re-localization to intracellular stores of cell surface-associated epithelial proteins, i.e., E-cadherin, rather than transcriptional repression. Interestingly, carcinoma cells utilizing this program migrate as clusters (also termed collective migration) in contrast to single-cell migration patterns observed in the traditional, transcriptionally defined EMT mechanisms [62,63]. In particular, various BC and colorectal cancer cell lines utilize this alternative program to undergo EMT. Emerging evidence suggests that pEMT can not only drive distinct modes of cell migration, but also enhances the E/M plasticity of cancer cells as well as cell fate plasticity [60] (Figure 1 and Figure 2). Previous work on EMT and CSCs suggested that stemness markers acquired during initial EMT are lost in the course of a cEMT in contrast to the maintenance of a stem-like phenotype after pEMT [64,65]. Moreover, a hybrid EMT phenotype is crucial for basal BC cell tumorigenicity demonstrating enhanced stemness which is paralleled by elevated Snail expression and activation of Wnt signaling pathways [66]. Another interesting study by Pastushenko and coworkers [67] investigated the spectrum of EMT states occurring during EMT in skin squamous cell carcinoma and in mammary tumors. The pEMT states in cancer cells localized within different parts of a tumor were associated with differences in their transcriptional and epigenetic programs and metastatic potential [67]. It would be interesting to reveal whether these different hybrid EMT states also respond differently to chemotherapeutic drug treatment.

A prominent number of CTCs with mesenchymal properties are detectable in patient samples after chemotherapy correlating with progression of the disease [68]. The presence of tumor cells in the circulation has been correlated with the presence of metastases in multiple cancers [69]. Of note, when analysing the EMT phenotype of CTCs, most studies found a prognostically relevant association between the presence of CTCs with a pEMT or a mesenchymal phenotype [68,70,71,72,73,74] (Figure 1). For instance, in BC patients, mesenchymal cells were highly enriched in CTCs and their presence was associated with disease progression. Intriguingly, in an index patient, reversible shifts between the hybrid and mesenchymal states accompanied each cycle of response to therapy and intermittent disease progression [68].

Recent findings have shown that the biological impact of EMT depends on the dynamics of its transition [75]. Mathematical modeling and experimental analysis of the EMT induced by TGF-β revealed a non-linear hysteretic response with E-cadherin repression being tightly controlled by the strength of the miR-200/ZEB negative feedback loop. Hysteretic EMT transfers a memory state, enabling it to persist long after withdrawal of the initial stimuli. Interestingly, while both hysteretic and non-hysteretic EMT imparts similar morphological changes and invasive potential on cancer cells, only hysteretic EMT enhances the efficiency of (lung) metastasis. Moreover, cells that have undergone hysteretic EMT differentially express stem cell and extracellular matrix-related genes with significant prognostic value [75].

Several lines of evidence suggest that hybrid E/M states also exist in human tumors (Figure 1 and Figure 2). Cancer cells co-expressing E-cadherin and vimentin were found in invasive BC [76] and subsets of tumors co-expressing these two markers exhibited the worst disease-free survival and overall survival among all BC patients analyzed. Different degrees of EMT were detected in xenografts derived from lung, breast, and esophagus SCC (small cell carcinoma) patients [67]. Epithelial and mesenchymal cells even co-exist within the same clone in most tumors of Pten/Trp53-deficient mice [77], suggesting that the induction of pEMT is likely to be an inherent property of most clones. This is somehow at odds with the traditional view that EMT usually occurs at the invasive front in the tumor buds, the morphological surrogate of EMT featuring cellular plasticity [78]. Acquisition of an E/Mstate is facilitated by the expression of EMT-inducing TFs and the activation of adult stem cell programs, i.e., canonical Wnt signaling. Furthermore, transition from the highly tumorigenic E/M state to a less-tumorigenic fully mesenchymal phenotype, which can be achieved i.e., by forced expression of Zeb1, is accompanied by a switch from canonical to non-canonical Wnt signaling. Identifying the central regulators of the various phenotypic states may prove useful in designing new therapeutic approaches [79,80] that function by shifting cancer cells between distinct states along the E/M spectrum [66] (Figure 2).

The phenotype switching in cases of drug exposure or drug withdrawal is seen with many types of cancer. Particularly, transdifferentiation via EMT has been shown to be indispensable for resistance of BC- and PDAC-derived cells to cyclophosphamide and gemcitabine, respectively [81,82]. However, differences exist in exactly how cancer cells evade therapy, including EMT, acquiring properties of CSCs or transdifferentiation potential [16,36,83,84,85] (Figure 1). These related cellular programs are accompanied by (re-)initiation of abnormal development pathways, suggesting that plasticity-driven resistance to therapy is governed by similar molecular mechanisms [40,86]. Together, these findings indicate that reprogramming of cancer cells during EMT provides a suitable paradigm to examine cancer cell plasticity.

## 4. Factors Involved in Plasticity and Transdifferentiation

CSC plasticity is controlled by both cell-intrinsic (cell-autonomous) and extrinsic (non-cell-autonomous) factors. CSCs can be protected, maintained, and expanded in CSC niches (CSCN) which can be reversibly established by mediators such as prostaglandin E2 signaling [87] and various cell types including MSCs and CAFs [88,89,90] (Figure 1). Intrinsic factors encompass, for instance, DNA damage, somatic mutations, epigenetic regulation of DNA and histone modification, as well as alternative gene splicing. Extrinsic components include the TME, injury, inflammation, viral infections, and drug treatment. A wide array of growth factors and their signaling pathways is involved in regulating cell plasticity, such as bone morphogenetic proteins, fibroblast growth factor, hepatocyte growth factor, Notch, platelet derived growth factor, sonic hedgehog, TGF-β, Wnt/β-catenin, and vascular endothelial growth factor [50].

### 4.1. Oncogenes, Tumor Suppressor Genes and Homeobox Transcription Factors

The ability for cell state reprogramming could be acquired through oncogenic (gain-of-function) mutations, i.e., in KRAS, which may cause constitutive signaling, loss-of-function mutations/loss-of-heterozygosity (LOH) in tumor suppressor genes, i.e., *APC*, *RB*, *TSP53*, *PTEN*, *BRCA*, or through epigenetic mechanisms [91]. Specifically, over-activation of stem cell signaling pathways such as Wnt/β-catenin, Notch, Shh, EGF, or TGF-β accompanied by oncogenic mutations, or LOH of tumor suppressor genes, can lead over time to carcinogenesis [92]. Tumor suppressors like P53 or PTEN have also been associated with CSC plasticity [93,94,95]. The combined loss of P53/PTEN in clonal prostate epithelial cells caused transformation of multipotent progenitors and led to EMT [96]. Moreover, mutations in *KRAS* and *APC* are also linked to the generation of stem-like cells [91]. Several studies have highlighted the importance of pluripotency-associated TFs, such as OCT3/4, SOX2, NANOG and KLF4 in modulating the generation of CSCs and cellular plasticity [97,98,99,100]. In glioblastoma multiforme (GBM), a core set of neuro-developmental TFs (POU3F2, SOX2, SALL2, OLIG2) has been identified that was sufficient to reprogram differentiated glioblastoma cells to CSCs [101]. Another study in GBM elaborated the complex interplay between genetic drivers and gain or loss of specific genes such as *CDK4*, *EGFR*, *PDGFR* and *NF1* along with cues from the TME in determining different cellular states exhibiting cell plasticity [102]. Cancer cell reprogramming can also be promoted by inhibition of tumor suppressor proteins via mutations or epigenetic silencing. For instance, retinoblastoma 1 (RB1) protein directly binds to the promotors of *POU5F1*, *SOX2*, and *NANOG* to repress their activities and loss of RB1 function therefore promotes reprogramming [91]. *TP53* and *PTEN* inactivation is crucial for resistance to abiraterone and progression from adenocarcinoma to castrate-resistant prostate cancer (CRPC) with neuroendocrine differentiation (CRPC-NE) by transdifferentiation [103]. An important contribution to cellular properties and tissue development is mediated by a variety of homeobox genes, including *PAX4* and *PDX1* (pancreas), *NKX2.1* (lung), *NKX3.1* (prostate), *CDX2* (colon, intestine), or *SOX2* (pancreas, prostate). Their aberrant expression or deregulation combined with oncogenic mutations has been demonstrated under conditions of chronic inflammation, injury, metaplasia and may contribute to reprogramming and plasticity in cancers [92]. Alterations in TF programs involved in embryonic development can also mediate tumor plasticity. For example, in murine models, concurrent loss of the lung lineage-specifying TF, Nkx2.1, from alveolar but not airway epithelium along with mutant Kras results in reprogramming of alveolar cells to mucinous adenocarcinomas similar to those of gastric or intestinal origin [104]. Likewise, in human non-small cell lung carcinomas (NSCLC) downregulation of NKX2.1 is associated with tumors resembling various gut tissues. These findings reveal a complex interplay of homeobox genes and oncogenes in driving cell plasticity and tumorigenesis [104]. In addition, the data demonstrate that aberrant tumor cell plasticity can reflect the normal developmental history of organs in that cancer cells acquire cell fates associated with developmentally related or adjacent organs [104].

### 4.2. Epigenetic Deregulation in EMT and Cell Plasticity

During distinct steps of the metastatic process, cancer cells experience dynamic and reversible transitions between epithelial and mesenchymal states/phenotypes, which are associated with changes in plasticity. These are enabled by transcriptional and epigenetic regulation of epithelial and mesenchymal genes. A large number of studies support the crucial role of epigenetic alterations in both the induction of EMT [105] and the generation of CSCs [59]. Chromatin modifiers such as histone deacetylases and/or DNA methyltransferases (DNMTs) can enhance plasticity, promote transition to partial E/M phenotypes, or stabilize heterochromatin configurations. Specific chromatin modifications catalyzed by histone deacetylases or polycomb group proteins, such as PRC2 (polycomb repressive complex 2) are involved in EMT, eventually resulting in a transcriptomic shift to a mesenchymal and stem-like phenotype [106]. Repression of epithelial genes is achieved by the enrichment of trimethylation at lysine-27 of histone H3 (H3K27me3) to form a bivalent modification with H3K4me3 to create a highly plastic and reversible state [107]. Furthermore, reduced trimethylation at lysine-4 (H3K4me3) facilitates the subsequent formation of the heterochromatic modification, H3K9me3, which is more stable and enhances the recruitment of DNMTs. DNA methylation on the epithelial gene promoters creates methylated CpG dinucleotides that are highly stable and can be propagated over many cell generations [107]. For instance, Snail recruits PRC2 to repress E-cadherin expression through increasing H3K27me3 on the *CDH1* promoter [108]. Repressed enhancer regions also harbor monomethylation at lysine-4 (H3K4me1) with either the absence or presence of H3K27me3, whereas activated enhancers feature H3K4me1 and elevated acetylated lysine-27 in histone H3 (H3K27ac) [109].

The MET marker, GRHL2 (Grainyhead-like 2) is considered a prototype factor for regulation of the chromatin accessibility. It inhibits the repressive activities of EMT-TFs and/or epigenetic repressors such as the PRC2 complex, histone deacetylases (HDACs) and DNMTs at promoters and/or enhancers of epithelial genes [110]. GRHL2 controls epigenetic remodeling and E/M plasticity during the intermediate phases of EMT/MET [110]. The chromatin remodeling protein HMGA2 was reported to be upregulated in hybrid E/M and mesenchymal state tumor cells of the mouse prostate, as well as in human CRPC. Knockdown of HMGA2, or suppressing HMGA2 expression with histone deacetylase inhibitors, inhibited E/M plasticity and stemness in vitro and markedly reduced tumor growth and metastasis in vivo [111]. Moreover, ΔNP63, a member of the p53 family of tumor suppressors, promotes the entrance into pEMT in squamous cell carcinoma [67]. Binding motifs for TFs such as AP1, Ets, Runx, and Tead have been found to be enriched in transition states, suggesting the possibility that common TFs are required to induce chromatin remodeling of the intermediate state of EMT [67,112]. Finally, noncoding RNAs, like long noncoding RNAs and miRs are important players in regulating pEMT states. Overexpression of the lncRNA, HOTAIR (HOX transcript antisense intergenic RNA), maintained pEMT phenotypes and induced migratory activity in HCC (hepatocellular carcinoma) cells [113]. Upregulation of MYOSLID (myocardin-induced smooth muscle lncRNA, inducer of differentiation), was associated with the modulation of pEMT, resulting in metastasis and poor prognosis in head-and-neck squamous cell carcinoma [114]. An oncomiR, miR-151a, induced pEMT and migration in NSCLC cells [115], while a double-negative feedback loop between members of the miR-200 family and ZEB1 regulates the dynamic transition between distinct E/M states (see Section 4.3.).

ZEB TFs use epigenetic mechanisms for regulation of transcription. Specifically, the PXDLS motifs in both ZEB1 and -2 recruit epigenetic silencing complexes to enable down-regulation of ZEB target genes via repressive histone marks. TWIST1 and -2 can act as either transcriptional repressors by recruiting histone deacetylases or by inhibiting acetyltransferases, or as transcriptional activators. Epigenetic repression of target genes is also controlled by both Snail and Slug through binding of their C-terminal zinc-fingers to the E-box consensus motif CAGGTG [116]. In addition, the evolutionarily conserved and N-terminally located SNAG transactivation domain, recruits epigenetic silencing complexes and promotes repressive modifications to histones (e.g., H3K4me3) that contribute to silencing the expression of Snail or Slug target genes [116,117,118,119]. The TWISTs can also regulate transcription by interacting with several other TFs (MyoD, RUNX1, RUNX2, p53, NFkB). Moreover, Twist1 needs to induce Slug to suppress the epithelial branch of the EMT program and Twist1 and Slug act in concert to promote the mesenchymal arm of EMT and tumor metastasis [120]. The current knowledge of the epigenetic regulation in pEMT is still limited and require further studies at the single-cell level to provide a clearer picture.

### 4.3. EMT-Related Transcription Factors

EMT-TFs are important players in the control of plasticity and transdifferentiation through both transcriptional and epigenetic mechanisms. Genome-wide RNA arrays have shown that overexpression of Zeb1 up-regulated the transcription of neuronal-specific genes and down-regulated that of epithelial-specific genes [121]. Interestingly, Zeb1 also has a role in transdifferentiation of mouse embryo fibroblasts (MEF) into functional neurons. Zeb1 was up-regulated during the early stages of transdifferentiation and its knockdown dramatically attenuated the efficiency of this process, while Zeb1 overexpression increased it. Zeb1 not only rapidly promoted the functional maturation of induced neuron-like cells, but also induced MEF-derived neurons to form functional synapses in vivo following transplantation. Moreover, Zeb1 was required for stemness and metabolic plasticity of the cancer cells in a mouse model of PDAC [122]. Another EMTTF, Twist1, inhibited cancer cell plasticity, dissemination, and lung metastasis in a mouse model of (oncogene-induced) BC. Interestingly, in a subset of the tumor cells, Twist1 was required for the expression of other EMT-inducing TFs (Snail, Slug, Zeb2), which collaborated with Twist1 to induce pEMT, basal-like tumor progression, and metastasis [123].

Zeb1, Twist1, Snail, Slug, or treatment with TGF-β promote both tumorigenicity and stemness of cancer cells (Figure 2). For instance, Zeb1 strongly represses the miR-200 family, whose members are potent inducers of epithelial differentiation [122,124], consequently increasing cell plasticity and tumor progression in PDAC cells. More specifically, Zeb1 promoted expression of the CSC marker CD44 in PDAC and BC cells [125], while in the same cells, SNnail and Slug decreased E-cadherin and stimulated ALDH expression, along with increases in sphere and colony forming capacity [126,127,128]. Tongue SCC demonstrated similar characteristics, whereby overexpression of Snail was accompanied by EMT and CSC-like properties [129]. These findings indicate a close relationship of EMT initiation and development of CSC subtypes in various tumors although tumor stemness is independently regulated from EMT. For instance, down-modulation of Twist1 in benign skin tumors diminished proliferative capacity paralleled by elevated apoptosis, and inhibited tumor maintenance and progression independently of its function during EMT [130].

### 4.4. Therapy-Associated Plasticity

Tumor cells may harness plasticity as a survival mechanism to escape immunosurveillance and resist chemotherapy-induced death [131]. The observation that clones with resistance-conferring mutations can pre-exist within an individual tumor prior to drug exposure or are further selected during treatment was suggestive of a rare subpopulation of CSCs, or poorly differentiated cancer cells, that is intrinsically more refractory to various types of cancer therapies due to enhanced drug efflux activities and increased self-renewal potential. This subpopulation of CSCs is characterized by its tumor-initiating capacity and plays a crucial role in tumor heterogeneity, chemoresistance and tumor invasion. Their ability to adopt a dormant/quiescent/slow cycling state may mean that they persist throughout the clinical history of a cancer patient. Due to their metastatic dormancy [131] CSCs play a critical role in tumor recurrence [132]. During these long periods of time the CSCs eventually develop mutations, the acquisition of which is essential to evolve into clinically relevant drug-resistant cells [133,134].

Conversely, drug therapy itself can exert selective pressures on the tumor cells that affect tumor evolution. A study by Sharma and coworkers provides mechanistic insights into the modes of therapy-induced cellular plasticity and underscores the use of epigenetic inhibitors in targeting tumor development [135]. Using single-cell transcriptomics the authors observed a selection-induced increase of H3K27ac modifications in the chromatin of drug-resistant cells. The drug-induced adaptation was acquired upon the loss of SOX2, and a concomitant gain of SOX9, the latter of which was enriched at drug-induced H3K27ac marks. This strongly suggests that tumor evolution could be driven by stem cell-switch-mediated epigenetic plasticity.

#### 4.4.1. Cancer Cell Transdifferentiation in Response to Therapy

A further avenue to cancer cell plasticity is represented by transdifferentiation of cancer cells along the endothelial cell lineage to support tumor angiogenesis [136]. The capability to form tube-like structures was observed in a CD133-positive CSC subpopulation of triple-negative BC [137]. Moreover, in renal cell carcinoma the expression of CD133 and CD44 was associated with high CSC marker expression and angiogenic structures correlating with poor survival [138]. These findings show that CSCs may not only interconvert among their subpopulations but can also generate different kinds of non-CSCs with a differentiated phenotype (Figure 1).

Cancer cell reprogramming and phenotypic switches in response to drug exposure has likewise been observed in a subset of NSCLC patients treated with anti-EGFR targeted therapy. These tumors may eventually develop resistance and recur as a different phenotype, commonly SCLC [37,41]. Similar observations were made in CRPC, where adenocarcinoma of the prostate treated with inhibitors of the androgen receptor (AR) switches to a tumor with neuroendocrine phenotype [103,139,140]. This “neuroendocrine prostate cancer” (NEPC) or CRPC-NE is a subtype of PC that develops mainly via neuroendocrine transdifferentiation of prostate adenocarcinoma in response to AR inhibition therapy. This variant of the disease is characterized by an aggressive clinical course, very short responses to conventional therapy and poor prognosis [141]. Recent studies have highlighted the role of epithelial plasticity, including transdifferentiation to alternate cell lineages and EMT in the development of NEPC. Although the underlying mechanisms driving neuroendocrine differentiation in anti-EGFR-treated NSCLC and anti-hormone therapy-treated PC remains unclear, switches between different cell fates or acquisition of specific oncogenic mutations occur in response to AR blockade/during the evolution to NEPC. More specifically, lineage tracing studies have demonstrated that combined loss of P53, RB, and PTEN in cancer cells treated with targeted therapy is necessary for this transdifferentiation switch, i.e., adenocarcinoma to neuroendocrine (small cell phenotype) in both PC and lung tumors [103,139,140,142]. Loss of tumor suppressor genes may enable these tumor cells to reexpress developmental TFs [143] and to exhibit cell plasticity under selective pressure of potent AR inhibition (prostate) or EGFR inhibition (NSCLC). Interestingly, reciprocal regulation between Slug and AR transcriptional regulation and protein bioactivity, as well as Slug-AR complex formation plays an important role in accelerating the androgen-independent outgrowth of CRPC [144]. Of note, RNAi-mediated down-regulation of AR resulted in upregulation of tumor cell-derived CCL2, and continuous recruitment and enhanced infiltration of macrophages. These TAMs which predominantly represent M2-type macrophages contribute to an immunosuppressive TME and support EMT and an increase of stem/progenitor subpopulations eventually displaying resistance to androgen-deprivation therapy [145]. Thus, EZH2, an epigenetic regulator and repressor element-1 silencing transcription factor (REST), that are both involved in differentiation along a neuroendocrine phenotype and relay therapy resistance in PC or lung cancer, may represent promising molecular targets. Inhibition of their activity may be suitable to reverse this phenotypic transformation and regenerate or maintain the drug-susceptible state [146]. However, since prostate CSC transdifferentiation into neuroendocrine cells is associated with worse prognosis, elucidation of its mechanistic basis is urgently needed in order to therapeutically target this disease [147]. The neuroendocrine transdifferentiation process apparently represents an escape mechanism to resist targeted therapy. During this process the cancer cells gain TFs in the neuroendocrine pathway that contribute to AR-independent (prostate) or EGFR-independent (lung) growth and survival because it imparts tumors with a stem cell-like state [41,103,139,148].

Small cell neuroendocrine (SCN) cancers (SCNCs) are another aggressive cancer subtype. As for NEPC, transdifferentiation towards a SCN phenotype has been reported as a resistance route in response to targeted therapies. These convergent SCNCs have shared vulnerabilities—as revealed by drug sensitivity screens—that are found across unannotated SCN-like epithelial cases, small round blue cell tumors and hematological malignancies [149]. Finally, in GBM, the recurrent tumor exhibits a more aggressive behavior due to a phenotypic shift towards the mesenchymal subtype, a phenomenon termed proneural-mesenchymal transition [150].

A crucial issue regards the question of whether CSC (trans)differentiation is a reversible process. Even in prototypically hierarchical malignancies, such as acute myeloid leukemia (AML), it is not clear whether CSC differentiation is unidirectional rather than reversible. Interestingly, in murine and human models of AML, deletion of PU.1 (a lineage-determining TF), or withdrawal of established differentiation agents, like all-trans-retinoic acid (ATRA), caused some mature leukemia cells to retrodifferentiate and adopt a stem-like state with a clonogenic and leukemogenic potential. [151].

Interfering with CSC transdifferentiation through pharmacologic intervention may offer a promising complement of standard chemotherapy. Therapeutically targeting plasticity in cancer may be generally achieved via two different approaches. The first type of intervention would either block or reverse retrodifferentiation to prevent cancer cells from becoming metastatic and/or drug-resistant. The second approach would inhibit a signaling pathway utilized by cells that have undergone EMT to enter the circulation, survive therapy, or suppress the host immune system. Principally, either of these two EMT-directed strategies by itself could inhibit tumor malignancy but because neither one of them is intended to kill the cancer cells, these will eventually become resistant. From this it follows that the cancer cells with EMT phenotypes need to be eradicated rather than merely blocking or reversing their EMT phenotypes. Despite being an attractive goal, this has been hindered by the unavailability of drugs that selectively kill cancer cells with an EMT phenotype due to their inherent chemoresistance.

For example, a differentiation therapy approach using ATRA for the treatment of APL has shown some promise [152], although progressive resistance to monotherapy with ATRA emerges over a relatively short period of time, typically within 3–6 months [152]. This leukemia differentiates into mature granulocytes after ATRA treatment. Melanoma is one of the most studied cancers with respect to transdifferentiation of CSCs. It was reported that CSCs in melanoma form spheroids in culture and melanoma cells from these spheroids showed increased potential to form tumors in vivo after injection into mice [45]. Using varying types of conditioned media, melanoma spheroid cells can transdifferentiate into multiple cell lineages, such as melanocytes, adipocytes, chondrocytes, or osteocytes [153]. These studies highlight both the concept of CSCs in melanoma and the ability of melanoma CSCs to undergo transdifferentiation. It has been reported that a similar transdifferentiation process could be induced by treating melanoma CSCs by upregulation of PPARγ [154]. In line with these observations, PPARγ agonists, such as rosiglitazone, have also been found to induce cellular transdifferentiation in various malignancies, including BC [155], CML [156], GBM [157] and myxoid liposarcomas [158,159].

Other potential therapeutic directions to modulate transdifferentiation of CSCs include their conversion into quiescent, postmitotic cells. Using a well-established adipogenesis induction protocol, Ishay-Ronen and colleagues have provided strong evidence for plasticity of mesenchymal-like BC cells, which can be exploited for therapeutic purposes by forcing their transdifferentiation towards (postmitotic) functional adipocytes both in vitro and in vivo, rather than by killing the cells directly [160,161,162]. Of note, this transdifferentiation occurs only in cell lines with mesenchymal features as opposed to those with epithelial features. These findings resemble the results from earlier studies, which have shown that EMT-derived cells, much like MSCs, have the potential of transdifferentiation into multiple lineages, particularly mesoderm-derived osteoblasts, chondrocytes and adipocytes [163,164,165,166]. The higher plasticity of tumor cells with mesenchymal attributes (e.g., those exhibiting pEMT phenotypes) is due to the mechanistic connection and functional overlap between EMT programs and the CSC phenotype [40,160,161,162,167]. In their study, Ishay-Ronen et al., have employed TGF-β to induce EMT programming and to prime the cells for subsequent adipocyte transdifferentiation. However, this growth factor is known for its negative role in adipocyte differentiation [168,169] and represses the adipogenic transdifferentiation of EMT-derived BC cells by activating MEK/ERK signaling [160,161,162]. To get around this apparent conflict, the authors have combined a MEK inhibitor (Trametinib) with the adipogenesis inducer, rosiglitazone, in their adipogenic transdifferentiation therapy. This drug combination strongly promoted the direct lineage conversion of those tumor cells in a patient-derived xenograft model [160,161,162]. Intriguingly, this promotion was restricted to cancer cells with hightened plasticity and specifically to those located at the invasive front of the primary tumor, the region where EMT most frequently occurs [170] supposedly in a TGF-β-dependent manner (see above). It is interesting to note that this spatial and functional specificity targets only those cells for adipogenesis that are intrinsically more refractory to existing therapeutic approaches due to the mechanistic link between EMT, CSCs and drug resistance. In combining conventional with transdifferentiation therapy the former will efficiently kill the proliferative cancer cells that make up the bulk of the tumor, whilst the transdifferentiation therapy eradicates invasive cells in areas of tumor budding that escape conventional therapies due to the development of a retrodifferentiated EMT/CSC phenotype [160,161,162]. This trans- differentiation-based strategy may advance the preclinical proof-of-concept to successful clinical trials with BC patients.

Although, by definition, there are differences between ATRA-based and adipogenesis-based transdifferentiation therapies, APL cells share in common with EMT-derived carcinoma cells the high plasticity and (re)differentiation capability that on the one hand renders these cells drug-refractory but on the other hand also vulnerable to terminal differentiation through appropriate pharmacological stimuli. The examples discussed above would suggest that increasing cancer cell plasticity by enforcing CSC (trans)differentiation could be a promising therapeutic strategy for overcoming drug resistance. However, it is unavoidable that plasticity-targeted therapies will also face many challenges and risks. For example, considering the tumor-promoting role of adipocytes in BC, the transdifferentiation therapy-induced enrichment of these cancer-associated adipocytes in the TME may pose a risk of further supporting the growth and metastasis of residual cancer cells. Also, and as discussed above, transdifferentiation towards a neuroendocrine phenotype has been linked to aggressiveness and drug resistance.

#### 4.4.2. Targeting Cell Plasticity of Non-CSCs and CSC Transition

As can be inferred from the above sections, a single therapeutic approach to eradicate all CSCs may be insufficient [171]. Rather, one should focus on identifying and then eradicating in the CSC niche the dominant drivers of plasticity among CSCs and adjacent differentiated non-CSCs to assist CSC-targeted therapy [172]. Rather than anti-CSC therapy alone, combined approaches with differentiation or normalization therapies (e.g., ATRA, oncostatin M, rosmantuzumab, tranylcypromine analogs) might have the potential to increase the survival of a much greater number of patients.

## 5. Conclusions and Future Perspectives

A major challenge in cancer therapy is prediction of the clinical response and resistance to anti-cancer drugs on an individual basis. This is due to the ability of certain cancer cells to acquire genetic or epigenetic alterations spontaneously or in response to signals from the TME. The capacity of cancer cells to generate multiple types of tumor-propagating cells that are distinguishable by their positions along a spectrum of epithelial-to-mesenchymal, stem-to-differentiated and embryonic-to-mature cell states further enlarges this plasticity (Figure 1). In most cases, tumor cells can achieve a new phenotype without losing their original one, suggesting that phenotype switching between two functionally independent states is a complicated and multi-stage dynamic process involving several intermediate states. This reprogramming capability endows tumor cells with phenotypic plasticity, as exemplified by patients with lung cancer or PC [41,103,139,148]. These studies have demonstrated the importance of identifying tissue lineage in predicting response, sensitivity or resistance, of an individual tumor in the same way mutational signatures and biomarkers are used to inform clinicians on chemotherapy or targeted therapies. The ability of a cancer cell to change/reprogram in response to the applied drug as it progresses, further complicates the choice of the most appropriate therapy. Thus, there is an urgent requirement to understand interrelationships between stem cell, E/M, and tumor-associated reprogramming events to design novel therapeutic strategies that diminish cell state plasticity and minimize the development of tumor heterogeneity. Recent research has impressively documented that plasticity may also expose weaknesses in cancer cells that could be harnessed for therapeutic strategies to be developed. For instance, CSCs can potentially differentiate into cell lineages other than the original lineage from which the tumor arose. Therefore, the feasibility of mesenchymal-subtype/CSC transdifferentiation in a therapeutic setting has just been confirmed in a series of proof-of-concept preclinical studies and will hopefully proceed soon to the clinical trial stage. However, currently there is no FDA-approved cancer treatment regimen based on transdifferentiation. Nonetheless, considerable accumulating data point to the validity and potential of exploiting tumor heterogeneity and, specifically, cancer cell phenotypic plasticity to overcome therapy resistance.

## Abbreviations

APL	Acute promyelocytic leukemia
ATRA	All *trans*-retinoic acid
BC	Breast cancer
CAFs	Carcinoma-associated fibroblasts
CRPC	Castration resistant prostate cancer
CSCs	Cancer stem(-like) cells
CSCN	Cancer stem cell niche
CTCs	Circulating tumor cells
DNMTs	DNA methyltransferases
ECM	Extracellular matrix
E/M	Epithelial/mesenchymal
EZH2	Enhancer of zeste homolog 2
(p) or (c)EMT	(Partial) or (complete) epithelial-mesenchymal transition
GBM	Glioblastoma multiforme
LOH	Loss-of-heterozygosity
MET	Mesenchymal-epithelial transition
miR	Microrna
MEFs	Mouse embryo fibroblasts
MRD	Minimal residual disease
MSCs	Mesenchymal stroma/stem-like cells
NEPC	Neuroendocrine prostate cancer
NSCLC	Non-small cell lung cancer
PDAC	Pancreatic ductal adenocarcinoma
PHSP	Post-hybrid selection process
PDGF	Platelet-derived growth factor
SCC	Small cell carcinoma
Shh	Sonic hedgehog
TAMs	Tumor-associated macrophages
TFs	Transcription factors
TGF-β	Transforming growth factor-β
TME	Tumor microenvironment
TNF-α	Tumor necrosis factor-alpha
Zeb1/2	Zinc finger E-box-binding 1/2
